# Incidence of venous thromboembolism after standard treatment in patients with epithelial ovarian cancer in Korea

**DOI:** 10.1002/cam4.3797

**Published:** 2021-02-26

**Authors:** Wonkyo Shin, Sanghee Lee, Myong Cheol Lim, Jipmin Jung, Hak Jin Kim, Hyunsoon Cho

**Affiliations:** ^1^ Department of Cancer Control and Population Health National Cancer Center Graduate School of Cancer Science and Policy Goyang Republic of Korea; ^2^ Center for Gynecologic Cancer Research Institute and Hospital National Cancer Center Goyang Republic of Korea; ^3^ Division of Tumor Immunology Research Institute and Hospital National Cancer Center Goyang Republic of Korea; ^4^ Center for Clinical Trials Research Institute and Hospital National Cancer Center Goyang Republic of Korea; ^5^ Cancer Big Data Center National Cancer Control Institute National Cancer Center Goyang Republic of Korea; ^6^ Branch of Cardiology Department of Internal Medicine National Cancer Center Goyang Republic of Korea; ^7^ Division of Cancer Registration and Surveillance National Cancer Center Goyang Republic of Korea

**Keywords:** cardiotoxicity, cumulative incidence, electronic health records, epithelial ovarian cancer, hospital‐associated complication, venous thromboembolism

## Abstract

**Background:**

Venous thromboembolism (VTE) is a hospital‐associated severe complication that may adversely affect patient prognosis. In this study, we evaluated the incidence of VTE and its risk factors in patients with epithelial ovarian cancer (EOC).

**Methods:**

We retrospectively analyzed the electronic health record data of 1268 patients with EOC who received primary treatment at the National Cancer Center, Korea between January 2007 and December 2017 to identify patients who developed VTE. Demographic, clinical, and surgical characteristics of these patients were ascertained. Competing risks analyses were performed to estimate the cumulative incidence of VTE according to the treatment type. The associations between putative risk factors and the incidence of VTE were evaluated using the Fine–Gray regression models accounting for competing risks of death.

**Results:**

VTE was the most prevalent cardiovascular event, found in 9.6% (*n* = 122) of all patients. Of these VTE events, 115 (94.3%) occurred within 2 years of EOC diagnosis. Advanced cancer stage at diagnosis (distant vs. localized, hazards ratio [HR])= 14.49, *p* = 0.015) and extended hospital stay (≥15 days, HR =3.87, *p* = 0.004) were associated with the incidence of VTE. There was no significant difference in the cumulative incidence of VTE between primary cytoreductive surgery followed by adjuvant chemotherapy and neoadjuvant chemotherapy followed by interval cytoreductive surgery (HR =0.81, *p* = 0.390).

**Conclusions:**

Approximately 10% of patients with EOC were diagnosed with VTE, which was the most common cardiovascular disease found in this study. The assessment of VTE risks in patients with advanced‐stage EOC with an extended hospital stay is needed to facilitate adequate prophylactic treatment.

## Graphical abstract

Approximately 10% of epithelial ovarian cancer (EOC) patients are diagnosed with venous thromboembolism (VTE) within two years. The incidence of VTE in EOC patients did not differ significantly between treatment options, namely, primary cytoreductive surgery (PCS) followed by adjuvant chemotherapy and neoadjuvant chemotherapy (NAC) followed by an interval cytoreductive surgery.

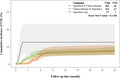

## 1 INTRODUCTION

Epithelial ovarian cancer (EOC) is a rare gynecologic malignancy. However, it is the leading cause of gynecologic malignancy‐related death in western countries.^1^ Similarly, in South Korea, the incidence of ovarian cancer continues to increase and is associated with the highest mortality rate among all gynecologic cancers.^2^, ^3^, ^4^ The standard treatment for ovarian cancer is either primary cytoreductive surgery (PCS) followed by adjuvant chemotherapy or neoadjuvant chemotherapy (NAC) followed by interval cytoreductive surgery (ICS).^5^, ^6^

During the course of treatment of ovarian cancer, several complications can develop.^7^ In most cases, there is no notable effect; however, some complications, such as cardiopulmonary complications and/or infection, can delay the standard cancer treatment. Furthermore, these complications may adversely affect patient prognosis.^8^, ^9^ Risk models have been developed in an attempt to predict these complications,^10^, ^11^ and algorithms have been created to reduce them.^12^ The correlations between cancer and cardiovascular diseases have also been the subject of previous studies.^13^, ^14^ Among cancer treatment‐related cardiovascular events, venous thromboembolism (VTE) is a severe hospital‐associated complication; however, more than half of VTE events are considered preventable with appropriate prophylaxis.^15^, ^16^, ^17^, ^18^

Recently, a report analyzed the risk of VTE following PCS and strategies to reduce this risk.^19^ However, in recent years, NAC followed by ICS was introduced as the standard of care, with efficacy that was the same as that of PCS and adjuvant chemotherapy. The risk of VTE could be associated with both chemotherapy and surgery; therefore, it is necessary to consider the risk factors for and incidence of VTE during the standard treatment period. In this study, we aimed to evaluate the incidence of VTE and its corresponding risk factors by analyzing the demographics and different treatments in patients with ovarian cancer diagnosed with VTE.

## 2 MATERIALS AND METHODS

### 2.1 Data sources

We obtained data from the Clinical Research Data Warehouse (CRDW), a database of electronic medical records created at the National Cancer Center (NCC), Korea in 2018. This CRDW database consists of medical records of patients with cancer. It contains over 2000 tables, primarily including information regarding cancer registration, clinic, nursing evaluation, surgery, and medications administered.^20^, ^21^ The CRDW assigns each patient the same anonymous identification key across all tables using a de‐identification system. Our data were extracted from several different tables, including the hospital‐based cancer registry (enrollment and demographics), clinic (outpatient and inpatient), nursing evaluation (past disease history and vitals), treatment (surgery, chemotherapy, and radiotherapy), and transfusion tables. The study was approved by the institutional review board of the NCC (NCC2019‐0025).

### 2.2 Study population

**FIGURE 1:**
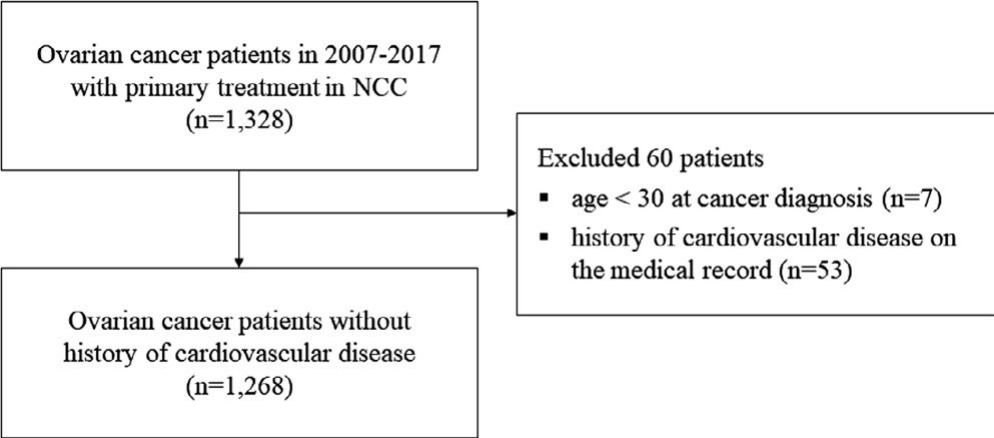
Patient selection flow chart

We identified 1328 patients who were newly diagnosed with ovarian cancer between January 2007 and December 2017 (International Classification of Diseases, Tenth Revision [ICD‐10] diagnosis codes C48.1, C56, and C57) from the cancer registration data. Patients who had received primary treatment at the NCC within 4 months of their cancer diagnosis were included. The following participants were excluded: (1) patients aged <30 years at the time of cancer diagnosis (*n* = 7) because the incidence of EOC below 30 years of age is very low^22^ and (2) patients with a history of cardiovascular disease (*n* = 53). The final cohort included 1268 patients with ovarian cancer (Figure 1) and was followed up from the day of EOC diagnosis until 31 December 2018, for cardiovascular diseases and all‐cause mortality.

### 2.3 Measurements

The incidences of nine different cardiovascular diseases, namely, secondary hypertension (ICD‐10: I15), ischemic heart disease (I20‐I25), VTE (I26, I80.2, and I82), pericardial effusion and cardiac tamponade (I31.3 and I31.9), cardiomyopathy due to chemotherapeutic agents and heart failure (I42.7 and I50), atrial fibrillation and flutter (I48), cerebrovascular disease (I60–I69), arterial embolism and thrombosis (I74), and others (occlusion and stenosis of the carotid artery [I65.2] and dissection of the aorta [I71.0]), were calculated. Further analysis was performed regarding the incidence of VTE events (I26, I80.2, and I82) within 2 years of EOC diagnosis. Data related to patients’ age; the Surveillance, Epidemiology, and End Results (SEER) stage; pathology; and primary treatment (yes/no) were extracted from the cancer registration data. Data regarding cancer treatment contain information on the therapy type, for example, surgery, chemotherapy, and radiotherapy. Detailed information on cancer treatment, including treatment dates, type (surgery type and drugs), and specific data (site of surgery and duration of surgery), was also extracted. Information on the treatment was reviewed for 2 years after EOC diagnosis or before the diagnosis of treatment‐related cardiovascular events within the 2 years. We checked whether patients undergoing surgery for ovarian cancer received blood transfusion within a week after surgery. Body mass index (BMI), the Eastern Cooperative Oncology Group (ECOG) performance status, and the incidence of hypertension, depression, and diabetes mellitus at the time of hospitalization were extracted from the nursing evaluation data. If there were multiple nursing evaluations, the one closest in time to the EOC diagnosis but prior to VTE onset was used.

### 2.4 Statistical analyses

Demographic, clinical, and surgical characteristics of patients with ovarian cancer in the VTE and non‐VTE groups were compared using *t*‐tests and chi‐squared tests. Competing risks analysis was performed to account for death prior to VTE development (*n* = 105, 10.5%). A total of 1003 patients who underwent surgery were included in the analysis, excluding those with "unknown" SEER stage cases at cancer diagnosis (*n* = 16, 1.6% among 1019 surgical patients). The cumulative incidence of VTE was estimated. Gray's test was used to compare the probabilities of VTE development according to the cancer treatment type over 24 months. The Fine–Gray competing risks regression models were fitted to estimate covariates’ effects on the subdistribution hazard function.^23^ The covariates evaluated for association with VTE were age at cancer diagnosis, the SEER stage, treatment at the NCC, hypertension, diabetes mellitus, dyslipidemia, site of surgery, duration of surgery, admission day, and transfusion. For all analyses, *p* < 0.05 was considered statistically significant. SAS 9.4 (version 9.4 SAS Institute Inc., Cary, NC, USA) was used for the analyses.

## 3 RESULTS

The overall incidences of cardiovascular diseases in 1268 patients are summarized in Table S1. Of these, 164 patients were diagnosed with cardiovascular diseases following EOC diagnosis, of whom 122 (74.4%) were diagnosed with VTE. Of the patients diagnosed with VTE, 115 (94.3%) were diagnosed within 2 years after their EOC diagnosis. Baseline characteristics and EOC treatments of patients in the VTE and non‐VTE groups are summarized in Table 1. Age, the ECOG performance status, and prior disease history (hypertension, diabetes, and depression) were not significantly different between the two groups. Stages were more advanced, and the proportion of patients who received chemotherapy alone was higher in the VTE group than in the non‐VTE group.

**TABLE 1:** Demographic and clinical characteristics of patients with ovarian cancer stratified according to the development of venous thromboembolism^a^

	VTE patients (*n* = 115) *n* ^b^ (%)	Non‐VTE patients (*n* = 1153) *n* ^b^ (%)	*p* value
Age at diagnosis (years), mean (SD)	56.1 (11.0)	54.2 (11.3)	0.1012
BMI (kg/cm^2^), mean (SD)^c^	23.2 (2.9)	23.1 (3.3)	0.7626
Age at diagnosis (years)			0.3447
<65	88 (76.5)	925 (80.2)	
≥65	27 (23.5)	228 (19.8)	
ECOG			0.8575
0	70 (66.7)	652 (67.9)	
1	30 (28.6)	254 (26.5)	
2+	—	54 (5.6)	
SEER stage			0.0001
Localized	—	185 (16.0)	
Regional	11 (9.6)	114 (9.9)	
Distant	102 (88.7)	833 (72.2)	
Unknown	—	21 (1.8)	
Operation			0.0004
No	37 (32.2)	212 (18.4)	
Yes	78 (67.8)	941 (81.6)	
Chemotherapy			0.5024
No	7 (6.1)	54 (4.7)	
Yes	108 (93.9)	1099 (95.3)	
Radiation			0.0207
No	114 (99.1)	1083 (93.9)	
Yes	—	70 (6.1)	
Treatment in NCC^d^			0.0032
PCS only	7 (6.1)	54 (4.7)	
Chemotherapy only	37 (32.2)	212 (18.4)	
PCS plus adjuvant chemotherapy	44 (38.3)	560 (48.6)	
NAC plus ICS	27 (23.5)	327 (28.4)	
Hypertension			0.1991
No	76 (66.7)	811 (72.3)	
Yes	38 (33.3)	310 (27.7)	
Depression			0.8489
No	103 (90.4)	1002 (89.8)	
Yes	11 (9.6)	114 (10.2)	
Diabetes mellitus			0.3699
No	99 (86.8)	933 (83.6)	
Yes	15 (13.2)	183 (16.4)	

Note

Results are reported as *n* (column %) unless otherwise noted, and cases ≤5 were marked as “—” sign.

Abbreviations: BMI, body mass index; ECOG, Eastern Cooperative Oncology Group performance status; ICS, interval cytoreductive surgery; IQR, interquartile range; NAC, neoadjuvant chemotherapy; NCC, National Cancer Center, Korea; PCS, primary cytoreductive surgery; SD, standard deviation; SEER, Surveillance, Epidemiology, and End Results; VTE, venous thromboembolism.

^a^ VTE diagnosed within 2 years of cancer diagnosis.

^b^ Due to the missing values (<16.8%), the sample size was smaller than the total for some variables.

^c^ BMI was missing for two patients in the VTE group and for 51 patients in the non‐VTE group.

^d^ Patients received radiation therapy were included in each treatment category (*n*): PCS only (1), chemotherapy only (12), PCS plus adjuvant chemotherapy (31), and NAC plus ICS (27).

Regarding surgical factors that were possibly related to the risk of VTE, more patients received blood transfusions within a week of surgery in the VTE group than in the non‐VTE group (84.6% vs. 73.8%, *p* = 0.036), and extensive surgery was performed in the VTE group (other: spleen, liver, etc.; 52.6% vs. 36.6%, *p* = 0.021). Furthermore, the duration of surgery (≥5 h, 80.8% vs. 66.9%, *p* = 0.012) and that of admission (≥15 days, 93.6% vs. 73.5%, *p* < 0.01) were longer in the VTE group than in the non‐VTE group (Table 2).

**TABLE 2:** Surgical characteristics of patients with ovarian cancer stratified according to the development of venous thromboembolism^a^

	VTE patients (*n* = 78)	Non‐VTE patients (*n* = 925)	*p* value
	*n* (%)	*n* (%)	
Duration of surgery (hours)^b^, mean (SD)	6.9 (2.6)	6.1 (2.5)	0.0069
Admission day, mean (SD)	27.8 (19.1)	20.2 (12.2)	0.0008
Blood transfusion			0.0356
No	12 (15.4)	242 (26.2)	
Yes	66 (84.6)	683 (73.8)	
Surgery area			0.0208
TAH, BSO, omentectomy, LND, else	10 (12.8)	159 (17.2)	
Bowel	27 (34.6)	427 (46.2)	
Other (spleen, liver, etc.)	41 (52.6)	339 (36.6)	
Duration of Surgery (hours)^b^			0.0116
<5	15 (19.2)	306 (33.1)	
≥5	63 (80.8)	618 (66.9)	
Length of admission (days)			<0.0001
<15	—	245 (26.5)	
≥15	73 (93.6)	680 (73.5)	

Note

Results are reported as *n* (column %) unless otherwise noted, and cases ≤5 were marked as “—” sign.

Abbreviations: BSO, bilateral salpingo‐oophorectomy; LND, lymph node dissection; SD, standard deviation; TAH, total abdominal hysterectomy; VTE, venous thromboembolism.

^a^ 1003 patients who underwent surgery were included in the analysis (unknown SEER stage at cancer diagnosis were excluded, *n* = 16, 1.6%).

^b^ Duration of surgery was missing for one patient in the non‐VTE group.

**FIGURE 2:**
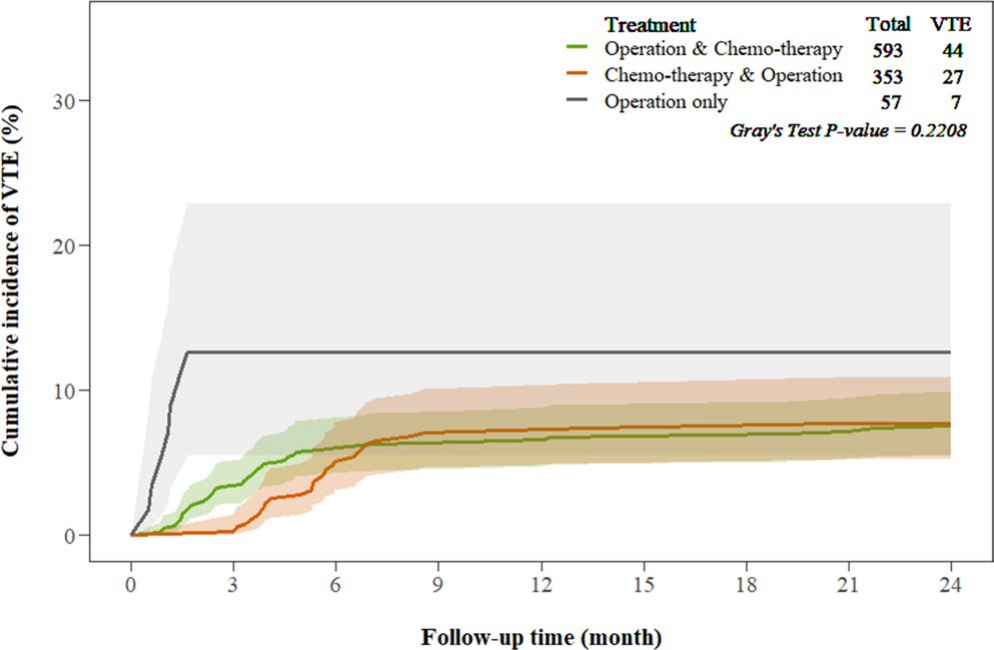
Cumulative incidence of venous thromboembolism in patients with ovarian cancer stratified by treatment type*. ICS, Interval cytoreductive surgery; NAC, Neoadjuvant chemotherapy; PCS, Primary cytoreductive surgery; VTE, venous thromboembolism. *:Shaded areas represent 95% confidence intervals

The cumulative incidence of VTE over the 2‐year period is shown in Figure 2. Regardless of the treatment type, the incidence of VTE sharply increased in the first 9 months, whereas VTE was rarely diagnosed after that. The cumulative incidence of VTE was 3.4% at 3 months, 5.9% at 6 months, 6.3% at 9 months, and 6.4% at 12 months in patients who underwent PCS plus adjuvant chemotherapy, whereas it was 0.3% at 3 months, 5.1% at 6 months, and 7.1% at 9 and 12 months in those who received NAC plus ICS. In patients who underwent PCS only, the cumulative VTE incidence was 12.6% at 3 months, and no VTE was diagnosed subsequently. The difference in the incidence of VTE between patients who underwent PCS plus adjuvant chemotherapy and those who received NAC plus ICS was not significant (*p* = 0.221).

Risk factors were analyzed in patients who underwent surgery. Univariate analysis revealed that the advanced SEER stage (distant vs. localized, HR =12.9, *p* = 0.0106), transfusion history (HR =1.88, *p* = 0.0448), long duration of surgery (≥5 h, HR =1.96, *p* = 0.0197), and prolonged admission (≥15 days, HR =4.91, *p* = 0.0006) were significantly associated with the incidence of VTE. After multivariate analysis, the SEER stage (distant vs. localized, HR =14.49, *p* = 0.0145) and duration of admission (≥15 days, HR =3.87, *p* = 0.0040) were identified as independent risk factors for VTE diagnosis (Table 3). The risk of VTE was approximately 14.5 times higher in patients diagnosed with advanced‐stage cancer than in those diagnosed with localized‐stage cancer, and the risk was four times higher in patients with a more extended hospital stay (≥15 days).

**TABLE 3:** Competing risk analysis for the development of venous thromboembolism in patients with ovarian cancer^a^.

Covariates	Univariable			Multivariable		
	Unadjusted HR	SE	*p* value	Adjusted HR	SE	*p* value
Age at diagnosis (years)						
<65	Reference			Reference		
≥65	1.54	0.26	0.1002	1.40	0.29	0.2430
SEER stage						
Localized	Reference			Reference		
Regional	14.46	1.04	0.0105	14.83	1.08	0.0128
Distant	12.92	1.00	0.0106	14.49	1.09	0.0145
Treatment in NCC^b^						
PCS plus adjuvant chemotherapy	Reference			Reference		
NAC plus ICS	1.00	0.24	0.9983	0.81	0.25	0.3898
PCS only	1.97	0.42	0.1045	5.82	0.50	0.0005
Past medical history/comorbidities						
Hypertension	1.29	0.25	0.2909	0.98	0.26	0.9353
Depression	0.87	0.40	0.7272	0.72	0.58	0.5759
Diabetes mellitus	0.85	0.33	0.6192	0.77	0.45	0.5625
Blood transfusion	1.88	0.31	0.0448	1.22	0.33	0.5485
Surgery area						
TAH, BSO, omentectomy, LND, else	Reference			Reference		
Bowel	1.00	0.37	0.9913	0.65	0.41	0.2946
Other (spleen, liver, etc.)	1.78	0.35	0.1037	0.96	0.41	0.9200
Duration of surgery (hours)						
<5	Reference			Reference		
≥5	1.96	0.29	0.0197	1.08	0.33	0.8141
Length of admission (days)						
<15	Reference			Reference		
≥15	4.91	0.46	0.0006	3.87	0.47	0.0040

Abbreviations: BSO, bilateral salpingo‐oophorectomy; HR, hazards ratio; ICS, interval cytoreductive surgery; LND, lymph node dissection; NAC, neoadjuvant chemotherapy; NCC, National Cancer Center, Korea; PCS, primary cytoreductive surgery; SE, standard error;SEER, Surveillance, Epidemiology, and End Results; TAH, total abdominal hysterectomy.

^a^ 1003 patients who underwent surgery were included in the analysis (unknown SEER stage at cancer diagnosis were excluded, *n* = 16, 1.6%).

^b^ Patients who received radiation therapy were included in each treatment category (*n*): PCS plus adjuvant chemotherapy (30), NAC plus ICS (27), and PCS only (1).

## 4 DISCUSSION

In patients with cancer, VTE is a severe hospital‐associated complication; however, more than half of VTE events are considered preventable with appropriate prophylaxis.^15^, ^16^, ^17^, ^18^ In our study, approximately 10% of patients with EOC developed VTE over a 2‐year treatment period. Wagner et al. also reported that the cumulative risk of VTE is around 10%; however, they analyzed only patients who underwent PCS.^19^ In contrast, our analysis also included patients who received ICS and NAC in addition to those who underwent PCS.

The exact mechanism of the increased risk of VTE in patients with ovarian cancer remains unknown; it is possible that metabolic changes in patients with cancer result in an increased incidence of VTE.^24^ Because tumor‐derived interleukin (IL)‐6 increases hepatic thrombopoietin synthesis and platelet production, as well as increases thrombotic events in patients with ovarian cancer, it has been suggested that interfering with the IL‐6‐thrombopoietin pathway may reduce VTE events.^25^

Although the incidence of VTE has increased rapidly in the Asian population in recent years, it is much lower than that in the western counterparts.^26^, ^27^ The incidence of VTE in the Korean population is reported to be approximately one‐eighth of that in the western population.^28^, ^29^ In addition, low‐molecular‐weight heparin (LMWH) has been found to be associated with postoperative bleeding complications in Korean patients.^30^, ^31^ The National Comprehensive Cancer Network (NCCN) and the American Society of Clinical Oncology (ASCO) guidelines recommend VTE prevention up to 4 weeks postoperatively in patients undergoing major abdominal or pelvic surgeries. A Cochrane systematic review has recently found that extended thromboprophylaxis (up to 28 days) with LMWH significantly reduces the risk of VTE compared with thromboprophylaxis during the in‐hospital period only, without increasing bleeding complications or mortality after major abdominal or pelvic surgeries.^32^ However, the Korean guidelines do not specify the duration of postoperative thromboprophylaxis.^33^ The Gynecologic Cancer Center of the NCC offers scheduled 7‐day LMWH treatment for prophylactic anticoagulation to all patients with ovarian cancer after surgery if they have no medical contraindication to anticoagulation therapy. Moreover, pneumatic compression stocking was applied to all patients. We administered 2500 IU of dalteparin sodium (Fragmin, Pfizer Ltd) subcutaneously from the next day of surgery for 7 days and monitored the blood coagulation parameters. This prophylactic regimen did not tend to increase the complication rate. Hospital stays in our institution are typically approximately 10 days, and the 28‐day regimen requires an outpatient setting that is somewhat challenging to apply in real clinical practice. This was also commented on in the NCCN and ASCO guidelines.

In our study, patients with advanced‐stage ovarian cancer and those who received chemotherapy alone had an increased rate of diagnosed VTE. Previous studies have found that chemotherapy is also an important risk factor for VTE in patients with cancer.^26^ Among the chemotherapeutic agents used for ovarian cancer, platinum (cisplatin/carboplatin)‐based regimens and bevacizumab have been found to increase the incidence of VTE.^34^, ^35^ According to the NCCN guidelines, NAC is indicated when an optimal cytoreductive surgery is unfeasible due to more advanced disease. Therefore, the probable reason why the incidence of VTE was high in the chemotherapy alone group was that the initial disease status was worse in this group than that in the PCS group. Thus, one interpretation is that many patients in the chemotherapy alone group did not undergo surgery because their disease progressed to more advanced or end‐stage during NAC.

In our study, the incidence of VTE increased in patients requiring a longer duration of surgery and hospital stay. Generally, the development of hospital‐associated complications during recovery is associated with more extended hospital stays, possibly leading to an increase in the incidence of VTE. A longer duration of surgery indicates that more extensive surgery was needed, or complications developed during surgery. The incidence of VTE was higher in patients with a transfusion history. Perioperative transfusion, including in the immediate postoperative period, increases the risk of VTE in gynecologic surgery.^36^, ^37^ Transfused red blood cells (RBCs) can cause inflammatory changes in patients’ hemodynamic systems.^38^ Furthermore, inflammation and hypercoagulation are linked, and RBC transfusion may lead to a hypercoagulable status.^39^

We did not find a significant difference in the incidence of VTE between patients who underwent PCS followed by adjuvant chemotherapy and those who received NAC followed by ICS. This result was similar to that previously reported in two large randomized clinical trials; in both studies, the incidence of VTE was approximately 2.5% in the PCS group and 0% in the NAC group.^5^, ^6^ However, those studies only reported postoperative adverse events and not the overall course of treatment, including chemotherapy. Another study also reported the incidence of VTE only during 6 months after surgery in patients undergoing PCS.^19^ In contrast with these reports, our study followed up all patients for more than 2 years regardless of the treatment course and found no overall difference between patients who underwent PCS followed by adjuvant chemotherapy and those who received NAC followed by ICS during that period.

There are several strengths to this study. This is the first study evaluating the incidence of VTE in patients with EOC during the standard treatment and observational period, including 2 years of follow‐up, which is essential for close monitoring of patients. We also included both standard treatments for ovarian cancer, namely, PCS followed by adjuvant chemotherapy and NAC followed by ICS. The risk factors associated with surgery, such as a longer duration of surgery and transfusion, were identified. We suggest that in patients with risk factors for VTE, active prophylactic treatment is needed through a multidisciplinary approach with cardiovascular and radiologic medical teams. Finally, our study used data from comprehensive electronic health records, including the accumulated records of patients with ovarian cancer over an 11‐year period (2007−2017). Therefore, this is one of the most extensive single institute study on cardiotoxicity in patients with ovarian cancer, specifically VTE, in the past decade.

There are some limitations to this study. First, this was a retrospective study; there was a lack of information about patients’ underlying diseases and demographic characteristics, which might have introduced some biases. Second, we did not use the International Federation of Gynecology and Obstetrics (FIGO) staging system, which is often used for the staging of gynecological cancers, because relevant information for FIGO staging was missing in a large proportion (>30%) of patients in the earlier years. Instead, we used the SEER stage information, and the results are expected to be comparable because both the SEER and FIGO staging represent the extent of the disease. Third, subclinical VTE might not have been included in the data because VTE was identified according to symptom‐related work‐up data rather than routine VTE screening. However, this study is the largest clinical study based on 11 years of accumulated comprehensive electronic health records of Korean patients with EOC. This study may prove to be an important source of information on VTE in Korean patients with ovarian cancer.

In conclusion, VTE increases medical costs, delays treatment, and worsens quality of life and directly affects survival in patients with cancer. Therefore, active prevention and treatment of VTE in patients with ovarian cancer is needed. In the future, selective VTE screening should be considered in patients with risk factors for VTE, as identified in this study, including advanced‐stage disease and prolonged admission. Also, this study showed that VTE was the most common cardiovascular disease in patients with EOC. To reduce the incidence of VTE, the precise evaluation and identification of the risk factors for VTE are needed, along with active prophylaxis. Predictive models to identify patients with EOC at risk for VTE must also be developed to ensure adequate prophylaxis in these patients.

## CONFLICT OF INTEREST

No potential conflicts of interest were reported by the authors.

## Supporting information

Table S1

## Data Availability

Data that support the findings of this study might be requested from the National Cancer Data Center (https://www.cancerdata.kr/main.do), and restrictions apply to the availability of these data. The data are not publicly available due to privacy or ethical restrictions.
