# Supplementary material for: Incidence of venous thromboembolism after standard treatment in patients with epithelial ovarian cancer in Korea
Source: Cancer Med. 2021 Feb 26;10(6):2045–53. doi: 10.1002/cam4.3797 (PMC7957187; doi:10.1002/cam4.3797)
Supplement: Supplementary file 1 — Table S1 [file CAM4-10-2045-s001.docx]

Supporting Table 1. Number of cardiovascular diseases occurrence in ovarian cancer patients *

|  | Follow-up time (year) | |
| --- | --- | --- |
| Types of Cardio vascular disease (ICD-10) † | ≤ 1 |  |
| Secondary hypertension (I15) | - | 6 |
| Ischemic heart disease (I20-I25) | - |  |
| Venous thromboembolism (I26, I80.2, I82) | 104 | 11 |
| Pericardial effusion, cardiac tamponade (I31.3, I31.9) | - | - |
| Cardiomyopathy due to chemotherapeutic agents, Heart failure (I42.7, I50) | - |  |
| Atrial fibrillation and flutter (I48) | - |  |
| Cerebrovascular disease (I60-I69) | 9 | - |
| Arterial embolism and thrombosis (I74) | - |  |
| Others ‡ | - |  |

* Cases ≤ 5 were omitted and marked as ‘-’ sign, and blank cells represent no case.

† Among total 1,268 patients, five patients had multiple cardiovascular diseases.

‡ Others include occlusion and stenosis of carotid artery (I65.2), dissection of aorta (I71.0).
